# A short-term intervention of ingesting iron along with methionine and threonine leads to a higher hemoglobin level than that with iron alone in young healthy women: a randomized, double-blind, parallel-group, comparative study

**DOI:** 10.1007/s00394-023-03213-w

**Published:** 2023-07-22

**Authors:** Yuko Tateishi, Sakiko Toyoda, Hitoshi Murakami, Ryo Uchida, Reiko Ichikawa, Takuya Kikuchi, Wataru Sato, Katsuya Suzuki

**Affiliations:** 1 Institute of Food Sciences and Technologies, Ajinomoto Co., Inc., Kanagawa, 210-8681 Japan; 2 Research Institute for Bioscience Products and Fine Chemicals, Ajinomoto Co., Inc., Kanagawa, 210-8681 Japan

**Keywords:** Iron absorption and utilization, Amino acids, Dietary nutrient, Hemoglobin, Young healthy women

## Abstract

**Purpose:**

Enhancing iron absorption and utilization is important for amelioration iron status faster and thereby, for improving quality of life. Dietary protein and amino acids, including methionine and threonine, have been reported to facilitate the absorption and utilization of dietary iron. Here, we investigated the effect of combined ingestion of methionine, threonine, and iron on the improvement of iron status during a short-term intervention, by comparing that with iron ingestion alone in healthy young women.

**Methods:**

This was a randomized, double-blind, parallel-group, comparative study with 45 participants (aged 20–39) randomly assigned to three groups (*n* = 15 each): one group was administered 200 mg methionine, 400 mg threonine, and 6 mg iron once daily (FEMT); another ingested 6 mg iron alone (FE); and the third group ingested a placebo (PCG). Blood samples and dietary nutrient data were collected before the intervention (week 0) and after 2, 4, and 6 weeks. Serum iron, hemoglobin, transferrin, and ferritin levels were measured.

**Results:**

Blood hemoglobin levels were significantly higher in the FEMT than in the FE group (*P* < 0.05) at week 4. Serum iron, transferrin, and ferritin levels were not changed across groups. In addition, our analyses showed that the observed increase in hemoglobin levels was affected by the intervention rather than changes in dietary nutrient intake.

**Conclusions:**

Ingestion of methionine and threonine with low doses of iron leads to a higher hemoglobin levels than that with iron alone in a short period of 4 weeks.

**Trial registration:**

University Hospital Medical Information Network Clinical Trial Registry (UMIN000046621).

**Supplementary Information:**

The online version contains supplementary material available at 10.1007/s00394-023-03213-w.

## Introduction

Iron deficiency is a common global health and nutritional problem. Although the condition is a concern in developing countries, iron deficiency also occurs in some developed countries, particularly among women and children. The World Health Organization estimates the anemia prevalence among non-pregnant women aged 15–49 to be 33% in low- and middle-income countries. While the prevalence is lower in high-income countries, in 2019, 14% of women in such countries were also anemic [1]. Iron deficiency without anemia, which refers to a decrease of iron storage in the body, is more common than iron deficiency anemia, which indicates a decrease of hemoglobin level. A survey in Japan showed that a quarter of the women in their 20s to 40s had iron deficiency anemia, whereas over half of them had iron deficiency without anemia (Ministry of Health, Labour, and Welfare, 2004–2008. National Health and Nutrition Survey Report). Iron deficiency has been reported to reduce the quality of life because it causes dizziness, shortness of breath, headache, reduced performance, and cognitive dysfunction [2–6]. Although iron supplementation is the standard treatment for iron deficiency anemia, high intakes should be avoided as they can lead to gastrointestinal side effects [7]. However, preventing and improving iron deficiency with small amounts of iron, such as that contained in food, is difficult because iron bioavailability is low. Several studies have shown that low-dose iron interventions (< 20 mg/day) failed to improve [8–10], or required a long time to treat (> 4 months) iron deficiency [11–15]. Therefore, to effectively prevent and eradicate iron deficiency, it is necessary to improve the absorption and utilization of dietary iron, which involves the synthesis of iron-containing proteins in the body such as hemoglobin, myoglobin, and iron-sulfur protein.

Dietary iron is mainly absorbed via transporters located on duodenal and upper jejunal enterocyte apical surfaces. Once iron enters the blood circulation, it binds to transferrin and is transported to tissues for iron-containing protein synthesis. Ferritin is an iron-storage protein that can be measured in serum. Low serum ferritin is indicative of iron store deficiencies. An imbalance between iron intake, utilization, and loss occurs when there is an iron deficiency. Ferritin and hemoglobin blood levels decrease with an increased transferrin level, measured as unsaturated iron-binding capacity (UIBC) and total iron-binding capacity (TIBC).

Some studies have suggested an important relationship between dietary iron intake and the prevalence of iron deficiency [16, 17]. Furthermore, other studies [18, 19] have demonstrated that dietary factors other than iron might be involved in iron status. To date, some nutritional studies have reported that vitamins can enhance iron absorption and utilization. For example, vitamin D, vitamin B12 and folic acid are required for erythropoiesis [16–18]. vitamin C increases non-heme iron absorption and modulates the iron uptake pathway in the cell [19]. Moreover intake of vitamin A [20, 21] is related to iron prevalence. In the systematic review of the human intervention studies, a combination of low doses of iron and vitamins showed that at least 8 weeks is required to improve iron deficiency in women and children [22]. For more rapid improvement of iron deficiency, other nutrients that have different functions than that of vitamins on iron status may be needed.

Previous studies have reported an association between dietary protein and iron deficiency. Protein deficiency causes reduced erythropoiesis [23] and serum transferrin level [24]. Amino acids, the components of protein, have also been reported to improve iron absorption via chelation [25–28], and erythroid cells need to consume a large amount of amino acids for producing hemoglobin [29]. Furthermore, blood concentration of branched-chain amino acids has been shown to positively correlate with the levels of hemoglobin and ferritin [30]. These findings suggested a possible link between some amino acids and iron absorption/utilization. Particularly, methionine and threonine, which are essential amino acids, have been shown to have potentially important functions in iron absorption and utilization by in vitro and in vivo studies. For example, these amino acids were suggested to improve iron absorption by chelating them [25, 31], and were shown to increase serum levels of erythropoietic hormones, such as erythropoietin [32] and IGF-1 [33, 34]. However, to the best of our knowledge, no human studies have focused on the effects of methionine and threonine on iron status.

We hypothesized that amino acids, particularly methionine and threonine, play a vital role for a rapid improvement of iron status, by enhancing the absorption and utilization of iron. In this randomized, double-blind, parallel-group, comparative study in healthy young women, we therefore aimed to understand the effect of ingestion of methionine and threonine in combination with low doses of iron on blood parameters related to iron status, during the short intervention period of 6 weeks, by comparing this to the ingestion of iron alone.

## Materials and methods

### Study design

This randomized, double-blind, parallel-group, comparative trial conducted in Tokyo examined blood iron parameters in healthy young women and administered one of the following for 6 weeks: methionine and threonine with iron (FEMT); iron alone (FE); or a placebo (PCG). The study was registered with the University Hospital Medical Information Network Clinical Trial Registry (UMIN000046621).

### Ethical approval

All participants provided informed consent before inclusion in the study. The study was conducted in accordance with the Declaration of Helsinki, and the protocol was approved by the Institutional Review Board and Ethics Committee of Ajinomoto Co., Inc. (no. AJI-001). A medical doctor who was not involved in the data analysis independently assessed the safety of the test food.

### Sample size

The sample size was calculated based on previous studies for significance in comparing FE and FEMT groups at 6 weeks of intervention [32, 35, 36]. Specifically, a randomized trial assessing dietary iron benefits among young women [35] provided a standard deviation estimate for a serum iron level change of 30.5 µg/dl. Standard deviations were assumed to be the same for all groups. The effect size was calculated in two steps. Firstly, the 6 mg iron intervention effect size was estimated to be 42 μg/dl based on a linear dose–effect relationship assumption and reported serum iron level changes in the same study. Secondly, a rat study [36] demonstrated that higher dietary protein increased iron absorption by approximately 1.5–1.8. In addition, suppementation of methionine and threonine to low protein diet doubled serum erythropoietin concentrations [32]. We estimated that compared to iron supplementation alone, the methionine, threonine, and iron intervention effect on iron absorption and utilization would be twice that of iron alone. The final effect size was assumed to be 42 μg/dl between FE and FEMT at 6 weeks of intervention. The estimated required sample size was 45 individuals (15 per group) using a two-sided test with a 5% significance level to achieve 80% statistical power based on the effect size and standard deviation mentioned above.

### Participants

**Fig. 1:**
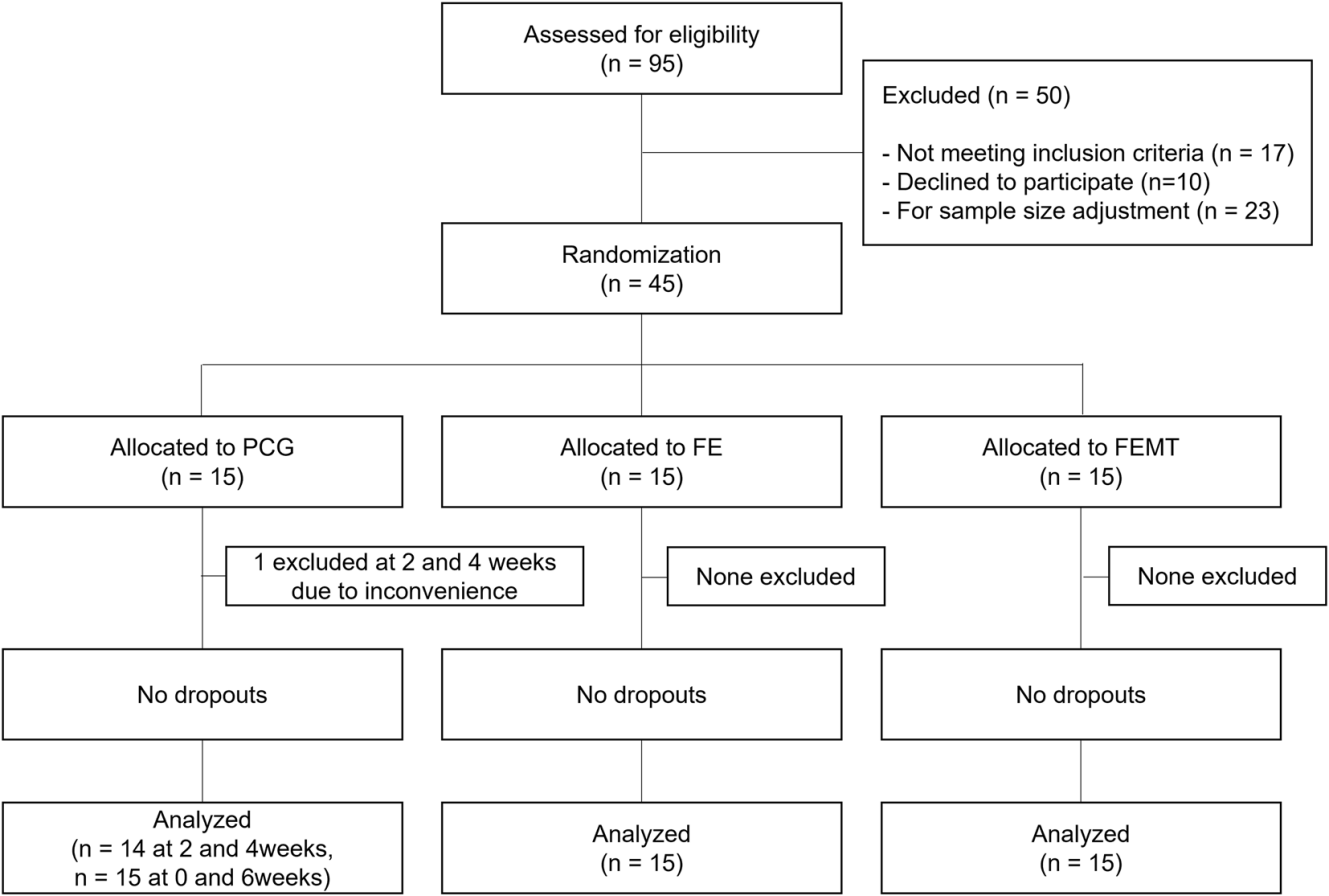
Consolidated standards of reporting trials (CONSORT) flow diagram. *PCG* placebo control group; *FE* intervention group receiving 6 mg iron alone once daily; *FEMT* intervention group receiving 200 mg methionine, 400 mg threonine, and 6 mg iron once daily

Figure 1 shows the Consolidated Standards of Reporting Trials (CONSORT) flow diagram. Healthy young women who had never been treated for iron deficiency, aged 20–39 years, were initially recruited and participated in the screening, for which blood samples were collected to be used in hematological and biochemical tests. In addition, dietary nutrient intake was determined using a food frequency questionnaire (FFQ) based on food groups [37]. The inclusion criteria were as follows: participants were included if they (1) were healthy and aged 20–39 years, (2) were able to use an electronic diary, and (3) fully understood the purpose of the study and volunteered to participate. Individuals were excluded if they (1) were treated for anemia, (2) required treatment for anemia based on comprehensive blood parameters as judged by a doctor, (3) consumed nutritional supplements or fortified foods, (4) frequently followed an extreme exercise routine that could cause hemolytic anemia, (5) reported high consumption of alcohol (over 60 g/day), (6) had a smoking habit, (7) had experienced excessive weight loss or weight gain, (8) were pregnant or breastfeeding, (9) had participated in other studies related to food, medicine, and cosmetics within 1 month, (10) had donated blood within 4 months, (11) were expected to change their lifestyle during the study period, for reasons such as planned travels, (12) felt uncomfortable providing blood, or (13) were judged to be inappropriate as participants by a doctor for other reasons such as menstrual irregularities. After the screening, 17 individuals were excluded. Subsequently, 10 individuals declined to participate and were excluded from the study as well. To match the pre-calculated sample size (*n* = 45), 23 additonal individuals who passed the screening test were excluded. The remaining 45 participants were randomly assigned to the three groups.

### Interventions

A survey demonstrated that Japanese women in their 20s consume 6.2 ± 2.5 mg (mean ± SD) of iron daily (Ministry of Health, Labour, and Welfare, 2019. National Health and Nutrition Survey Report). Thus, iron dosages were determined to be 6 mg/day to meet the Japanese iron Dietary Reference Intake (DRIs, 10.5 mg/day). Here, the first group was instructed to take two capsules containing 200 mg of methionine, 400 mg of threonine, and 6 mg of iron (57.7 mg of sodium ferrous citrate) with a cup of water once daily, 2 h after meals (FEMT group). The second group was instructed to take the same number of capsules containing 6 mg of iron simultaneously and in the same manner (FE group). The third group was instructed to consume a composition containing no amino acids or iron (PCG group). Methionine and threonine were supplied from Ajinomoto Co., Inc., and sodium ferrous citrate was supplied from Mitsubishi chemical co., ltd. All participants were asked to record their daily consumption. The duration of the intervention period was set at 6 weeks.

### Measures

Blood samples were collected at baseline (week 0) and after 2, 4, and 6 weeks of the intervention. In a previous study [35] that examined the effects of low-dose iron (9 mg/day) in young women, serum iron recovered after 6 weeks of intervention; however, other parameters did not recover. Therefore, in our study, serum iron levels were measured as the primary outcome, and hemoglobin, ferritin, unsaturated iron-binding capacity (UIBC), and total iron-binding capacity (TIBC) were measured as the secondary outcomes. One participant in the placebo group did not present for the blood collection at week 2 and 4.

Dietary nutrient intake during the intervention was calculated based on the participants’ food records covering 3 days prior to the blood collection at week 0, 2, 4, and 6, respectively. Energy and nutrient intake were automatically calculated using nutrition calculation software (Excel Eiyo-Kun ver. 9).

To evaluate physical conditions related to iron deficiency before and after the intervention, participants were scored based on the frequency of each of the following at week 0 and 6: (1) dizzy or lightheaded, (2) shortness of breath or palpitations, (3) headache, (4) feeling cold, (5) looking pale, (6) feeling exhausted, (7) waking up tired, (8) difficulty to concentrate, and (9) decreased motivation. The scores were defined as follows: 1 = never, 2 = 1–2 days in a week, 3 = 3–4 days in a week, 4 = 5–6 days in a week, and 5 = every day.

### Safety evaluation

Clinical laboratory measurements were taken before and after the intervention period to assess the safety of the composition of methionine, threonine, and iron. The following physical parameters were measured: height, body weight, body mass index (BMI), body fat mass, blood pressure, and pulse rate. In addition, the following blood parameters were measured: white blood cell count, red blood cell count, hemoglobin, hematocrit, platelet count, total protein, albumin, aspartate aminotransferase, alanine aminotransferase, lactate dehydrogenase, total bilirubin, alkaline phosphatase, γ-glutamyl transpeptidase, urea nitrogen, creatinine, uric acid, total cholesterol, low-density lipoprotein cholesterol, high-density lipoprotein cholesterol, triglyceride, fasting plasma glucose concentration, glycated hemoglobin, sodium, potassium, chloride, iron, zinc, and magnesium.

### Statistical analysis

The total study population was analyzed based on the intention-to-treat concept. The sample size for blood parameters in the PCG at week 2 and 4 were *n* = 14 due to some missed visits.

The following analyses were performed according to the pre-specified analysis plan. Serum iron, hemoglobin, UIBC, TIBC, and ferritin levels are expressed as the mean ± SD. Ferritin levels were log-transformed to obtain a normal distribution. The Tukey–Kramer test was applied to confirm that age, BMI, and blood parameters related to iron deficiency did not differ across the groups at baseline. Furthermore, to estimate the effects of the intervention on blood parameters, generalized linear mixed-effects models (GLMM) for repeated measures were calculated as follows:

1 $$y_{ijt} = \mu + {\text{Treatment}}_{j} + {\text{Time}}_{{t(0{\text{w}},2{\text{w}},4{\text{w}},6{\text{w}})}} + {\text{Time}}_{t} \times {\text{Treatment}}_{j} + \left( {1|{\text{Subject}}_{i} } \right) + \varepsilon_{ijt}$$

The outcome variable (*y*) in (1) indicates blood parameters at week 0, 2, 4, and 6. Treatment refers to the FE, FEMT, and PCG interventions. FE was set as the intercept of treatment because the comparison between FE and FEMT was the primary focus of this study. Time indicates 0, 2, 4, and 6 weeks of intervention. The model was fitted with random intercepts for participant ID.

To analyze changes in hemoglobin levels, we subtracted the initial hemoglobin level (at week 0) from the values at week 2, 4, and 6. Differences in changes in hemoglobin level across the three groups were compared using the Tukey–Kramer test. Dietary nutrient intake is expressed as the median ± interquartile range. The Mann–Whitney *U* test with Holm correction was applied to compare differences in dietary nutrient intake across the three groups. Distributions of physical condition scores at baseline (week 0) and after 6 weeks of intervention in each group were compared using Fisher’s exact test.

Pearson’s correlation coefficients and *P*-values were estimated post hoc to investigate changes between baseline and 6-week hemoglobin levels in the FEMT group. Fixed effects were estimated post hoc with a GLMM adjusted for vitamin D, to confirm the robustness of the intervention effects on hemoglobin levels considering differences in dietary vitamin D intake.

All statistical analyses were performed using R version 4.0.2 (R Foundation for Statistical Computing, Vienna, Austria), and statistical significance was set at *P* < 0.05.

## Results

### Compliance

Compliance with the interventions was recorded at 100% for 36 participants, 97.6% for 3, 95.2% for 4, 92.9% for 1, and 85.7% for 1 participant. No dropouts due to health issues related to this study were recorded.

### Participant characteristics

Table 1 shows the characteristics of the participants at baseline (week 0). No significant differences in blood parameters were observed across the groups. The average hemoglobin level was 13.1 ± 0.9 g/dl, which is comparable to the average value for Japanese women (National Health and Nutrition Survey in Japan, 2019). The median dietary nutrient intake at baseline calculated using the food record method for vitamin C and folic acid was lower than the Dietary Reference Intakes in Japan (DRIs: 100 mg/day and 240 µg/day, respectively). However, the median intake of these nutrients determined at the screening test using a FFQ, which is a better-validated method for calculating nutrient intake, was in line with the DRIs (data not shown). Thus, the sufficiency of the intake levels of these nutrients remains inconclusive. On the other hand, nutrient intake based on both food records and the FFQ indicated that energy, vitamin D, retinol, and iron intakes were lower than the DRIs, suggesting that the everyday meals of the participants provided insufficient levels (2000–2050 kcal/day, 8.5 µg/day, 650–700 µgRAE/day, and 10.5 mg/day, respectively).

**Table 1:** Baseline characteristics of all included participants

Parameters^a^	PCG (*n* = 15)	FE (*n* = 15)	FEMT (*n* = 15)
Age (y)	30.1 (5.8)	31.3 (3.7)	30.3 (5.5)
BMI (kg/m^2^)	21.6 (2.0)	21.6 (2.3)	21.4 (1.9)
Parameters related to iron status^b^			
Serum iron (μg/dl)	114.5 (64.1)	125.9 (57.4)	115.1 (57.3)
Hemoglobin (g/dl)	13.2 (0.9)	13.3 (0.9)	12.9 (0.8)
UIBC (μg/dl)	260.0 (95.8)	224.9 (84.3)	232.7 (66.9)
TIBC (μg/dl)	374.5 (50.5)	350.8 (47.9)	347.9 (41.9)
Log ferritin (ng/ml)	4.1 (1.2)	4.3 (1.1)	4.1 (1.3)
Nutrient intake			
Energy (kcal/day)	1496 (517)	1196 (485)	1343 (445)
Protein (g/day)	58.9 (23.4)	47.4 (14.8)	51.2 (14.4)
Fat (g/day)	55.1 (27.0)	40.3 (19.1)	48.0 (8.5)
Carbohydrates (g/day)	193.4 (62.0)	172.6 (59.1)	192.0 (58.8)
Iron (mg/day)	6.0 (1.9)	5.5 (2.6)	5.2 (1.9)
Retinol (μgRAE/day)	146 (120.8)	106.3 (78.5)	140.4 (65.4)
Vitamin B12 (μg/day)	3.6 (5.2)	3.4 (5.0)	3.3 (2.6)
Folic acid (μg/day)	204.1 (102.2)	189.5 (100.1)	177.4 (81.2)
Vitamin C (mg/day)	58.9 (34.0)	51.4 (32.2)	49.8 (49.1)
Vitamin D (μg/day)	3.1 (4.8)	1.6 (3.0)	2.1 (5.7)

^a^Age, BMI, and iron deficiency parameters are expressed as the mean (SD). Nutrient intakes calculated based on food records are expressed as the median (interquartile range)

^b^*BMI* body mass index; *UIBC* unsaturated iron-binding capacity; *TIBC* total iron-binding capacity

### Intervention effects

The blood parameters related to iron status assessed during the study are presented in Table 2. No significant difference was observed in the primary outcome, serum iron levels, across the three groups. As for the secondary outcomes, estimates from the GLMM showed that hemoglobin levels were significantly higher in the FEMT than in the FE group at week 4 (*P* = 0.023). This suggests that the ingestion of methionine and threonine with iron can increase hemoglobin levels compared to the ingestion of iron alone. Ferritin levels were significantly lower in the PCG than in the FE group at week 6; however, no significant difference was observed between the FE and the FEMT group. The changes in hemoglobin levels from baseline are presented in Table 3. At 4 and 6 weeks of intervention, hemoglobin levels were lower in the PCG and higher in the FEMT group compared to baseline, resulting in significant differences between these two groups. No significant difference in hemoglobin level changes was observed between the PCG and the FE group at any intervention point. In the FEMT group, we observed a negative correlation between hemoglobin levels at baseline and observed changes at 6 weeks of intervention (*r* =  − 0.63, *P* = 0.01).

**Table 2:** Changes in iron status during the experimental period

Parameter	Week	PCG^a,b^ (*n* = 14–15)	FE^a^ (*n* = 15)	FEMT^a^ (*n* = 15)	Estimate, 95% CI, *P*_(Week × Treatment)_^c^	
					FE vs. FEMT	FE vs. PCG
Serum iron	0	114.5 (64.1)	125.9 (57.4)	115.1 (57.3)		
	2	87.3 (28.8)	98.7 (49.7)	86.1 (30.6)	−1.87, [−39.49, 35.76], 0.922	0.18, [−37.86, 38.23], 0.992
	4	83.5 (33.8)	117.3 (46.7)	106.4 (42.9)	−0.13, [−37.76, 37.49], 0.994	−22.20, [−60.24, 15.84], 0.251
	6	83.4 (33.4)	93.5 (42.8)	100.8 (36.2)	18.0, [−19.63, 55.63], 0.346	1.20, [−36.43, 38.83], 0.950
Hemoglobin	0	13.2 (0.9)	13.3 (0.9)	12.9 (0.8)		
	2	12.8 (0.9)	13.3 (0.8)	13 (0.8)	0.01, [−0.42, 0.44], 0.976	−0.41, [−0.85, 0.03], 0.065
	4	12.8 (1.0)	12.9 (1.2)	13.1 (0.7)	0.50, [0.07, 0.93], 0.023*	−0.14, [−0.58, 0.29], 0.523
	6	12.7 (1.0)	13.1 (0.9)	13.2 (0.6)	0.36, [−0.07, 0.79], 0.101	−0.31, [−0.74, 0.12], 0.162
UIBC ^d^	0	260.0 (95.8)	224.9 (84.3)	232.7 (66.9)		
	2	285.0 (69.6)	244.9 (78.0)	264.5 (50.7)	11.87, [−28.66, 52.39], 0.564	1.52, [−39.52, 42.55], 0.942
	4	282.6 (66.5)	219.7 (76.4)	243.4 (71.0)	15.87, [−24.66, 56.39], 0.441	24.22, [−16.81, 65.25], 0.246
	6	288.3 (62.6)	243.8 (82.4)	247.6 (41.0)	−4.00, [−44.53, 36.53], 0.846	9.47, [31.06, 49.99], 0.645
TIBC ^d^	0	374.5 (50.5)	350.8 (47.9)	347.9 (41.9)		
	2	372.3 (55.0)	343.5 (54.2)	350.6 (41.5)	10.00, [−5.87, 25.87], 0.215	1.36, [−14.71, 17.43], 0.868
	4	366.1 (49.3)	337.0 (53.8)	349.8 (45.6)	15.73, [−0.13, 31.60], 0.052	1.68, [−14.40, 17.75], 0.837
	6	371.7 (47.4)	337.3 (55.8)	348.4 (38.9)	14.00, [−1.87, 29.87], 0.083	10.67, [−5.20, 26.53], 0.186
Log ferritin	0	4.1 (1.2)	4.3 (1.1)	4.1 (1.3)		
	2	4.0 (1.1)	4.6 (0.9)	4.2 (1.0)	−0.27, [−0.69, 0.15], 0.212	−0.32, [−0.75, 0.10], 0.138
	4	4.2 (1.2)	4.4 (0.9)	4.3 (1.1)	0.11, [−0.31, 0.53], 0.616	0.09, [−0.34, 0.52], 0.683
	6	4.1 (1.1)	4.8 (1.2)	4.4 (1.1)	−0.25, [−0.68, 0.17], 0.235	−0.50, [−0.93, −0.08], 0.019*

Bold values represent the significantly different (*P < 0.05, ** *P* < 0.01)

^a^All values are expressed as the mean (SD). *PCG* placebo control group; *FE* intervention group receiving 6 mg iron alone once daily; *FEMT* intervention group receiving 200 mg methionine, 400 mg threonine, and 6 mg iron once daily

^b^The sample size in the PCG at week 2 and 4 was *n* = 14 due to missed visits

^c^Estimates and *P*-values were obtained from the generalized linear mixed models constructed using blood parameters as outcome variables, and week (0, 2, 4, and 6) and treatments (FE, FEMT, and PCG) as explanatory variables. The test statistics are marked with *P < 0.05. *CI* confidence interval

^d^*TIBC* total iron-binding capacity; *UIBC* unsaturated iron-binding capacity

**Table 3:** Changes in hemoglobin level from baseline

Parameter (vs. baseline)	Week	PCG^a,b^ (*n* = 14–15)	FE^a^ (*n* = 15)	FEMT^a^ (*n* = 15)	*P*^c^		
					FEMT vs FE	FEMT vs PCG	FE vs PCG
Hemoglobin	2	−0.4 (0.5)	0 (0.7)	0 (0.4)	0.999	0.064	0.069
	4	−0.5 (0.5)	−0.3 (0.6)	0.2 (0.6)	0.054	0.007**	0.657
	6	−0.4 (0.6)	−0.1 (0.5)	0.2 (0.6)	0.215	0.008**	0.323

Bold values represent the significantly different (*P < 0.05, ** *P* < 0.01)

^a^All values are expressed as the mean (SD). *PCG* placebo control group; *FE* intervention group receiving 6 mg iron alone once daily; *FEMT* intervention group receiving 200 mg methionine, 400 mg threonine, and 6 mg iron once daily

^b^The sample size in the PCG at week 2 and 4 was *n* = 14 due to missed visits

^c^The Tukey–Kramer test was used for comparison between the three groups. The test statistics are marked with **P < 0.01

Dietary nutrient intake recorded during the study is presented in Table 4. Only vitamin D intake, which has been reported to be effective in improving iron deficiency, differed between the FE and the FEMT groups at week 4. The GLMM for estimating the effect of the intervention on hemoglobin levels (as shown in Table 2) was further adjusted for dietary intake of vitamin D (Table 5) to examine the influence of dietary vitamin D intake on hemoglobin levels. The results show that the effect of FEMT treatment at week 4 was significant even after adjustments for vitamin D intake.

**Table 4:** Dietary nutrient intake during the study period

Nutrient intake	Week	PCG^a^ (*n* = 15)	FE^a^ (*n* = 15)	FEMT^a^ (*n* = 15)	*P*^b^		
					FEMT vs FE	FEMT vs. PCG	FE vs. PCG
Energy (kcal)	0	1496 (517)	1196 (485)	1343 (445)	0.268	0.567	0.466
	2	1365 (531)	1309 (223)	1387 (416)	1.000	1.000	1.000
	4	1549 (418)	1373 (540)	1467 (618)	1.000	1.000	1.000
	6	1507 (386)	1256 (420)	1448 (481)	0.411	0.902	0.411
Protein (g)	0	58.9 (23.4)	47.4 (14.8)	51.2 (14.4)	0.899	0.899	0.899
	2	53.3 (22.2)	55.8 (17.1)	48.0 (16.1)	1.000	1.000	1.000
	4	57.0 (9.6)	51.8 (15.0)	52.7 (26.3)	1.000	1.000	0.736
	6	48.9 (20.5)	46.5 (15.7)	49.7 (17.5)	1.000	1.000	0.521
Fat (g)	0	55.1 (27.0)	40.3 (19.1)	48.0 (8.5)	0.442	0.442	0.304
	2	46.5 (26.4)	35.9 (18.1)	50.1 (14.1)	0.439	0.653	0.619
	4	47.2 (32.7)	50.2 (21.9)	51.3 (31.4)	1.000	1.000	1.000
	6	52.7 (22.2)	40.2 (12.6)	48.5 (28.4)	0.148	0.967	0.099
Carbohydrate (g)	0	193.4 (62.0)	172.6 (59.1)	192.0 (58.8)	1.000	1.000	1.000
	2	179.0 (68.3)	190.9 (54.5)	183.1 (55.3)	1.000	1.000	1.000
	4	184.1 (42.2)	190.7 (61.5)	187.6 (68.0)	1.000	1.000	1.000
	6	191.4 (52.6)	175.4 (82.8)	187.2 (50.0)	1.000	1.000	1.000
Iron (mg)	0	6.0 (1.9)	5.5 (2.6)	5.2 (1.9)	0.814	0.814	0.814
	2	5.6 (2.8)	5.8 (2.5)	5.2 (2.3)	1.000	1.000	1.000
	4	6.1 (2.4)	5.8 (1.8)	5.5 (2.6)	1.000	1.000	1.000
	6	6.7 (2.8)	5.3 (1.7)	6.0 (2.5)	0.927	0.927	0.927
Retinol (µgRAE)	0	146 (120.8)	106.3 (78.5)	140.4 (65.4)	0.403	0.806	0.378
	2	128.9 (51.0)	121.6 (74.0)	112.2 (88.8)	1.000	1.000	1.000
	4	131.4 (150.9)	145.9 (89.3)	166.1 (85.5)	1.000	1.000	1.000
	6	127.1 (77.8)	123.0 (111.5)	199.4 (125.4)	0.146	0.146	0.852
Vitamin B12 (µg)	0	3.6 (5.2)	3.4 (5.0)	3.3 (2.6)	1.000	1.000	1.000
	2	3.8 (3.9)	3.5 (3.8)	2.9 (2.5)	1.000	1.000	1.000
	4	3.0 (2.5)	2.6 (3.3)	3.6 (5.2)	0.244	0.244	0.819
	6	3.0 (2.1)	4.0 (1.9)	3.3 (5.2)	1.000	1.000	0.374
Folic acid (µg)	0	204.1 (102.2)	189.5 (100.1)	177.4 (81.2)	1.000	1.000	1.000
	2	178.1 (84.3)	180.6 (151.6)	176.0 (71.2)	1.000	1.000	1.000
	4	213.6 (105.4)	237.6 (138.6)	195.0 (107.2)	1.000	1.000	1.000
	6	204.8 (152.4)	188.8 (88.9)	202.2 (102.1)	1.000	1.000	1.000
Vitamin C (mg)	0	58.9 (34.0)	51.4 (32.2)	49.8 (49.1)	1.000	1.000	1.000
	2	33.1 (42.8)	47.5 (42.6)	60.0 (39.1)	0.713	0.411	0.534
	4	64.9 (50.7)	66.8 (38.1)	47.7 (38.8)	0.348	0.825	0.825
	6	47.1 (52.9)	49.6 (24.5)	61.5 (36.1)	1.000	1.000	1.000
Vitamin D (µg)	0	3.1 (4.8)	1.6 (3.0)	2.1 (5.7)	0.595	0.950	0.595
	2	1.8 (2.4)	3.3 (4.1)	2.7 (2.0)	1.000	1.000	1.000
	4	1.8 (2.4)	1.7 (1.2)	2.4 (2.9)	0.017*	0.186	0.547
	6	2.0 (3.9)	3.2 (4.1)	2.5 (4.9)	0.958	0.958	0.958
Methionine (g)	0	1.21 (0.51)	0.94 (0.34)	1.01 (0.42)	1.000	1.000	1.000
	2	0.99 (0.50)	1.07 (0.42)	0.99 (0.35)	1.000	1.000	1.000
	4	1.26 (0.47)	1.00 (0.37)	1.02 (0.54)	1.000	1.000	0.749
	6	1.05 (0.51)	1.03 (0.27)	0.94 (0.53)	1.000	1.000	1.000
Threonine (g)	0	2.08 (0.86)	1.65 (0.58)	1.70 (0.62)	1.000	1.000	1.000
	2	1.70 (0.93)	1.92 (0.77)	1.70 (0.56)	1.000	1.000	1.000
	4	2.17 (0.68)	1.82 (0.57)	1.80 (0.99)	1.000	1.000	0.605
	6	1.79 (0.87)	1.78 (0.47)	1.62 (0.92)	1.000	1.000	1.000

Bold values represent the significantly different (*P < 0.05, ** *P* < 0.01)

^a^All values are expressed as the median (interquartile range). *PCG* placebo control group; *FE* intervention group receiving 6 mg iron alone once daily; *FEMT* intervention group receiving 200 mg methionine, 400 mg threonine, and 6 mg iron once daily

^b^The Mann–Whitney *U* test with Holm correction was used for comparison between the three groups. The test statistics are marked as *P < 0.05

**Table 5:** Effect of vitamin D intake on hemoglobin levels

	Estimate, 95% CI, *P*_(Week × treatment)_^a^	
	FE vs FEMT^b^	FE vs PCG^b^
Week 2 × treatment	< 0.00, [−0.43, 0.43], 0.989	−0.43, [−0.87, 0.01], 0.053
Week 4 × treatment	0.52, [0.09, 0.95], 0.019*	−0.14, [−0.58, 0.29], 0.512
Week 6 × treatment	0.36, [−0.07, 0.79], 0.100	−0.33, [−0.76, 0.10], 0.130

Bold values represent the significantly different (*P < 0.05, ** *P* < 0.01)

^a^The crude model (analyzed in Table 2) was adjusted for vitamin D intake, and the effect of treatment on hemoglobin levels was estimated. The test statistics are marked as *P < 0.05

^b^*PCG* placebo control group; *FE* intervention group administered 6 mg iron alone once daily; *FEMT* intervention group administered 200 mg methionine, 400 mg threonine, and 6 mg iron once daily

Physical condition scores at baseline and at 6 weeks of intervention are presented in Supplemental Table 1. Scores for “looking pale” differed between the groups at baseline, however, after 6 weeks of intervention, no differences in physical condition scores were observed between the groups.

### Safety evaluations

No abnormal fluctuations were observed in clinical laboratory values, urine laboratory test results, vital data, height, weight, or BMI, indicating the absence of any safety concerns. Safety was assessed through medical interviews with each participant, and no adverse effect related to the interventions were reported. One participant did not present for the blood collection at intervention week 2 and 4; however, this was not due to side effects of the intervention.

## Discussion

In this study, we hypothesized that combined ingestion of methionine and threonine with iron increases iron absorption and utilization, resulting in the accelerated improvement of iron status during the short period of intervention. This study aimed to understand the effects of co-ingestion of methionine, threonine, and iron (FEMT) on blood parameters during the 6 weeks of the intervention, by comparing the effects with those of ingestion of iron alone (FE). Here, the primary outcome, serum iron level, was not different across the three groups. However, hemoglobin level, one of the secondary outcomes, was higher in the FEMT group than in the FE at 4 weeks of intervention. There was no significant difference between the FEMT and FE groups at 6 weeks of intervention; however, a similar trend of higher hemoglobin level in the FEMT group was also observed (*P* = 0.1, [GLMM]). Furthermore, hemoglobin changes from baseline were significantly higher in the FEMT group than FE at 4 and 6 weeks of intervention. As is known, the differentiation and maturation of red blood cells take approximately 1 month, and our findings, related to the hemoglobin level after 4 weeks, were reasonable in this respect.

The median dietary iron intake of our participants was lower than the dietary reference intakes (DRIs). Additionally, energy, vitamin D, and retinol intake, all of which are known to affect iron status [20, 21, 38–41], were also below the DRIs in our study sample. This suggests that participants were at a high risk of iron deficiency at baseline because of their dietary patterns. To clarify the effect of vitamin D intake on hemoglobin levels at 4 weeks of intervention, the GLMM further adjusted for vitamin D intake was constructed; however, we did not observe any effect of vitamin D intake on hemoglobin levels between the FE and the FEMT group. This result suggested that the increase in hemoglobin levels observed in the FEMT group was due to the intervention with methionine, threonine, and iron rather than to changes in nutrient intake. Note that the vitamin D effect on hemoglobin levels was inconclusive as its status can be influenced by other factors, including outdoor activities. Further analysis should be performed to confirm the association between vitamin D metabolite (e.g. 25(OH)D) levels and hemoglobin.

No significant effects were observed on blood parameters other than hemoglobin. Serum iron levels, the primary outcome of this study, were not affected by any intervention. Large intra- and inter-participant variations were observed, and serum iron levels increased from the screening test to week 0 of the intervention in many participants. While there is no clear explanation for this observation, serum iron levels may be influenced by the diurnal cycle [42] or dietary iron. This parameter might therefore not be an appropriate indicator to investigate the effect of our intervention. Serum ferritin levels, the indicator of iron storage, tended to be as low as 24.7 ± 22.3 ng/ml (mean ± SD), in our participants, of whom 24 had an iron deficiency without anemia (< 20 ng/ml of ferritin). During the study period, serum ferritin was significantly higher in the FE than in the PCG group at 6 weeks of intervention, but levels did not differ between the FE and the FEMT group. Whether the intervention had an effect on serum ferritin levels thus remains inconclusive. Although some previous studies of iron deficiency in female athletes without anemia reported that serum ferritin levels recovered after 6–8 weeks of intervention with 30 or 135 mg/day of iron [43, 44], we administered a reduced amount of iron (6 mg/day) to our participants. A longer intervention period may have been required to confirm the intervention effect on serum ferritin levels in our study. Serum TIBC, which indicates levels of total transferrin and increases during iron deficiency, tended to be higher in the FEMT than in the FE group at intervention week 4 and 6 (*P* = 0.052 and *P* = 0.083, [GLMM], respectively). However, no significant effect was observed on UIBC, indicates iron-free transferrin level (*P* = 0.441 and *P* = 0.846, [GLMM], respectively), and transferrin saturation (data not shown in the text; *P* = 0.877 and *P* = 0.549, [GLMM], respectively), at the same intervention points. This suggests that the increase in TIBC was not due to iron deficiency. Since dietary proteins and amino acids can accelerate transferrin synthesis [24], The level of transferrin might be affected by the intake of methionine and threonine.

Our study suggests that methionine and threonine may influence hemoglobin synthesis through various functions, including absorption, transport, and dietary iron utilization. While methionine and threonine are reported as iron-chelate amino acids [25], amino acid–iron complex formation in the intestinal environment may be difficult owing to significant chemical conditions, such as pH [45]. Thus, the main effect of methionine and threonine could not pertain to iron absorption. Compared to iron alone, methionine, threonine, and iron ingestion tended to increase serum TIBC without decreasing transferrin saturation, suggesting that these amino acids improve iron transport functioning. Furthermore, methionine and threonine ingestion have been reported to affect IGF-1 and erythropoietin blood levels, which are erythropoietic hormones [46]. Serum IGF-1, the growth hormone that has an anabolic effect on dietary protein [47], was increased in rats fed a high-methionine [48], and decreased in pigs fed a low threonine diet [33]. Additionally, erythropoietin blood levels decreased in rats fed a low-protein diet, and these levels increased with dietary methionine and threonine supplementation [32]. Although our study did not measure these blood hormone levels, methionine and threonine may affect hemoglobin levels through these functions. Vitamin C is known to improve iron bioavailability using mechanisms distinct from amino acids by increasing absorption. The methionine and threonine effect may be enhanced by combining other nutrients such as vitamin C.

Our study demonstrated the potential effect of combined ingestion of methionine and threonine with iron for rapid improvement in iron deficiency. To the best of our knowledge, no previous studies have shown that hemoglobin levels recover within 8 weeks after intervention with low doses of iron and other nutrients. However, some limitations should be noted. First, this study was conducted with healthy participants without anemia, and the effect of co-ingestion of methionine, threonine, and iron in women with iron deficiency anemia is unknown. Second, this study conducted exploratory with small size of participant. Larger study is needed to confirm the effect of methionine and threonine on hemoglobin level. Third, hemoglobin level in the PCG decreased during the study, which is probably due to the seasonal variation. Hemoglobin levels are reported to decrease with increasing temperatures in Japan [49], and our study was conducted from winter to spring when the temperature rises. The effects of co-ingestion of methionine and threonine in another season when hemoglobin levels rise, should be evaluated. Forth, further research is required to understand how methionine and threonine enhance the effect of dietary iron. Finally, in this study, participants ingested 6 mg of iron as sodium ferrous citrate, which contains ferrous iron (Fe^2+^) and has higher iron absorption than ferric iron (Fe^3+^) [50]. Thus, when combining methionine and threonine with ferric iron or other lower absorption compounds, it may require greater iron amounts to improve iron status faster.

## Conclusions

Co-ingestion of methionine and threonine with low doses of iron resulted in significantly increased hemoglobin level after a short intervention of 4 weeks compared with the ingestion of iron alone. Additionally, our analyses show that the observed increase in hemoglobin level was affected by the intervention, rather than from changes in dietary nutrient intake. Our results suggest that methionine and threonine effectively improve iron availability in young women, and that a combination supplementation with iron may help prevent and ameliorate iron deficiency.

## Supplementary Information

Below is the link to the electronic supplementary material.

Supplementary file1 (DOCX 30 KB)

## Data Availability

All data sets generated during and/or analyzed during the present study are not publicly available but are available from the corresponding author on reasonable request.
