# Supplementary material for: A short-term intervention of ingesting iron along with methionine and threonine leads to a higher hemoglobin level than that with iron alone in young healthy women: a randomized, double-blind, parallel-group, comparative study
Source: Eur J Nutr. 2023 Jul 22;62(7):3009–19. doi: 10.1007/s00394-023-03213-w (PMC10468430; doi:10.1007/s00394-023-03213-w)
Supplement: Supplementary file 1 — Supplementary file1 (DOCX 30 KB) [file 394_2023_3213_MOESM1_ESM.docx]

**European Journal of Nutrition**

Original Article

**A short-term intervention of ingesting iron along with methionine and threonine leads to a higher hemoglobin level than that with iron alone in young healthy women: A randomized, double-blind, parallel-group, comparative study**

Yuko Tateishi ^1*^, Sakiko Toyoda ^2^, Hitoshi Murakami ^1^, Ryo Uchida ^1^, Reiko Ichikawa ^1^, Takuya Kikuchi ^1^, Wataru Sato ^2^, and Katsuya Suzuki ^1^

^1^ Institute of Food Sciences and Technologies, Ajinomoto Co., Inc, Kanagawa, 210-8681, Japan;

^2^ Research Institute for Bioscience Products & Fine Chemicals, Ajinomoto Co., Inc, Kanagawa, 210-8681, Japan;

*****Correspondence: [yuko.tateishi.tq3@asv.ajinomoto.com](mailto:yuko.tateishi.tq3@asv.ajinomoto.com)

Supplementary Information

**Supplemental Table 1. Physical conditions related to iron deficiency at baseline and after 6 weeks of intervention.**

|  |  | **0 week** ^b^ | | | | |  | **6 weeks** ^b^ | | | | |  |
| --- | --- | --- | --- | --- | --- | --- | --- | --- | --- | --- | --- | --- | --- |
|  | **Treatment ^a^** | **1** | **2** | **3** | **4** | **5** | ***P*** ^c^ | **1** | **2** | **3** | **4** | **5** | ***P*** ^c^ |
| **Dizzy or lightheaded** | **PCG** | 11 (73%) | 2  (13%) | 2  (13%) | 0  (0%) | 0  (0%) | 0.713 | 12 (80%) | 2  (13%) | 1  (7%) | 0  (0%) | 0  (0%) | 0.637 |
|  | **FE** | 9  (60%) | 5  (33%) | 1  (7%) | 0  (0%) | 0  (0%) |  | 9  (60%) | 5  (33%) | 1  (7%) | 0  (0%) | 0  (0%) |  |
|  | **FEMT** | 11 (73%) | 2  (13%) | 1  (7%) | 1  (7%) | 0  (0%) |  | 11 (73%) | 4  (27%) | 0  (0%) | 0  (0%) | 0  (0%) |  |
| **Shortness of breath or palpitations** | **PCG** | 7  (47%) | 6  (40%) | 2  (13%) | 0  (0%) | 0  (0%) | 0.158 | 11 (73%) | 4  (27%) | 0  (0%) | 0  (0%) | 0  (0%) | 0.097 |
|  | **FE** | 8  (53%) | 6  (40%) | 1  (7%) | 0  (0%) | 0  (0%) |  | 5  (33%) | 9  (60%) | 1  (7%) | 0  (0%) | 0  (0%) |  |
|  | **FEMT** | 10 (67%) | 1  (7%) | 4  (27%) | 0  (0%) | 0  (0%) |  | 9  (60%) | 4  (27%) | 2  (13%) | 0  (0%) | 0  (0%) |  |
| **Headache** | **PCG** | 7 (47%) | 5 (33%) | 2 (13%) | 1 (7%) | 0 (0%) | 0.443 | 7 (47%) | 7 (47%) | 0 (0%) | 1 (7%) | 0 (0%) | 0.419 |
|  | **FE** | 7 (47%) | 3 (20%) | 5 (33%) | 0 (0%) | 0 (0%) |  | 4 (27%) | 9 (60%) | 2 (13%) | 0 (0%) | 0 (0%) |  |
|  | **FEMT** | 8 (53%) | 6 (40%) | 1 (7%) | 0 (0%) | 0 (0%) |  | 5 (33%) | 7 (47%) | 3 (20%) | 0 (0%) | 0 (0%) |  |
| **Feeling cold** | **PCG** | 2 (13%) | 1 (7%) | 5 (33%) | 4 (27%) | 3 (20%) | 0.513 | 3 (20%) | 5 (33%) | 7 (47%) | 0 (0%) | 0 (0%) | 0.165 |
|  | **FE** | 1 (7%) | 4 (27%) | 5 (33%) | 2 (13%) | 3 (20%) |  | 3 (20%) | 5 (33%) | 6 (40%) | 1 (7%) | 0 (0%) |  |
|  | **FEMT** | 3 (20%) | 3 (20%) | 2 (13%) | 6 (40%) | 1 (7%) |  | 7 (47%) | 3 (20%) | 2 (13%) | 3 (20%) | 0 (0%) |  |
| **Looking pale** | **PCG** | 11 (73%) | 1  (7%) | 3  (20%) | 0  (0%) | 0  (0%) | **0.013*** | 12 (80%) | 2  (13%) | 1  (7%) | 0  (0%) | 0  (0%) | 0.234 |
|  | **FE** | 15 (100%) | 0  (0%) | 0  (0%) | 0  (0%) | 0  (0%) |  | 13 (87%) | 2  (13%) | 0  (0%) | 0  (0%) | 0  (0%) |  |
|  | **FEMT** | 11 (73%) | 4  (27%) | 0  (0%) | 0  (0%) | 0  (0%) |  | 9  (60%) | 6  (40%) | 0  (0%) | 0  (0%) | 0  (0%) |  |
| **Feeling exhausted** | **PCG** | 5 (33%) | 4 (27%) | 3 (20%) | 3 (20%) | 0 (0%) | 0.319 | 4 (27%) | 8 (53%) | 2 (13%) | 1 (7%) | 0 (0%) | 0.769 |
|  | **FE** | 5 (33%) | 3 (20%) | 7 (47%) | 0 (0%) | 0 (0%) |  | 6 (40%) | 9 (60%) | 0 (0%) | 0 (0%) | 0 (0%) |  |
|  | **FEMT** | 7 (47%) | 3 (20%) | 2 (13%) | 3 (20%) | 0 (0%) |  | 5 (33%) | 7 (47%) | 2 (13%) | 1 (7%) | 0 (0%) |  |
| **Waking up tired** | **PCG** | 5 (33%) | 4 (27%) | 1 (7%) | 3 (20%) | 2 (13%) | 0.298 | 6 (40%) | 6 (40%) | 2 (13%) | 1 (7%) | 0 (0%) | 0.948 |
|  | **FE** | 4 (27%) | 6 (40%) | 5 (33%) | 0 (0%) | 0 (0%) |  | 6 (40%) | 8 (53%) | 1 (7%) | 0 (0%) | 0 (0%) |  |
|  | **FEMT** | 6 (40%) | 4 (27%) | 3 (20%) | 2 (13%) | 0 (0%) |  | 7 (47%) | 6 (40%) | 2 (13%) | 0 (0%) | 0 (0%) |  |
| **Difficult to concentrate** | **PCG** | 6  (40%) | 6  (40%) | 2  (13%) | 1  (7%) | 0  (0%) | 0.415 | 8  (53%) | 6  (40%) | 1  (7%) | 0  (0%) | 0  (0%) | 0.870 |
|  | **FE** | 10 (67%) | 2  (13%) | 2  (13%) | 1  (7%) | 0  (0%) |  | 10 (67%) | 4  (27%) | 1  (7%) | 0  (0%) | 0  (0%) |  |
|  | **FEMT** | 10 (67%) | 1  (7%) | 2  (13%) | 2  (13%) | 0  (0%) |  | 8  (53%) | 4  (27%) | 2  (13%) | 1  (7%) | 0  (0%) |  |
| **Decreased motivation** | **PCG** | 4  (27%) | 6  (40%) | 4  (27%) | 1  (7%) | 0  (0%) | 0.683 | 5  (33%) | 9  (60%) | 0  (0%) | 1  (7%) | 0  (0%) | 0.055 |
|  | **FE** | 8  (53%) | 4  (27%) | 1  (7%) | 2  (13%) | 0  (0%) |  | 10 (67%) | 5  (33%) | 0  (0%) | 0  (0%) | 0  (0%) |  |
|  | **FEMT** | 6  (40%) | 5  (33%) | 2  (13%) | 2  (13%) | 0  (0%) |  | 8  (53%) | 3  (20%) | 3  (20%) | 1  (7%) | 0  (0%) |  |

^a^PCG, Placebo control group; FE, Intervention group administered 6 mg iron alone once daily; FEMT, Intervention group administered 200 mg methionine, 400 mg threonine, and 6 mg iron once daily. The Fisher’s exact test was used for comparison between the three groups.

^b^The scores for physical conditions based on a questionnaire survey were defined as follows: 1 = never, 2 = 1–2 days a week, 3 = 3–4 days a week, 4 = 5–6 days a week, and 5 = every day. All values are expressed as the number and percentage (%).

^c^The Fisher’s exact test was used for comparison between the three groups. The test statistics are marked as *P < 0.05.
